# Digital Simulation of a Speech Therapist’s Professional Activity: Contribution to the Preservation and Promotion of Children’s Mental Health

**DOI:** 10.1192/j.eurpsy.2026.11832

**Published:** 2026-07-31

**Authors:** A. Akhmetzyanova, T. Artemyeva, Z. Egorova

**Affiliations:** Department of Psychology and Pedagogy of Special Education, Kazan Federal University, Kazan, Russian Federation

## Abstract

**Introduction:**

At the initial stages of professional training, future speech therapists primarily acquire theoretical knowledge, with limited opportunities to apply it in the diagnosis and correction of speech disorders. Previous studies indicate insufficient development of diagnostic competencies among graduates, as well as a lack of effective tools for their formation. The use of digital simulation of professional activities enables students to gain preliminary practical experience without direct interaction with children, thereby enhancing their readiness for real-world practice.

**Objectives:**

To present the development algorithm of a digital simulator that models the diagnostic activities of a speech therapist and to assess its potential for implementing a personalized approach in student training.

**Methods:**

A structured survey was conducted among 345 undergraduate and master’s students enrolled in the “Special (Defectological) Education” program at Kazan Federal University. The questionnaire covered the extent of diagnostic experience gained during internships, interest in digital educational tools, and preferred training formats. The proposed simulator consists of two modules: a **training module** (recognition of speech sound disorders) and a **diagnostic module** (accuracy assessment), both supported by two purpose-built video libraries — one featuring standard pronunciation samples and the other containing examples of various speech sound disorders.

**Results:**

Fifty-three percent of respondents reported encountering all major types of speech sound disorders during internships in educational institutions; 94% supported the creation of a digital simulator. Sixty-five percent preferred a blended learning format: preliminary virtual training followed by in-person practice. The simulator development process includes three key stages: (1) training participants to imitate various speech sound disorders, (2) creating a comprehensive video library of documented disorders, and (3) developing and hosting the digital simulator, which enables viewing video cases and completing diagnostic assessments. Anticipated benefits include expanding the range of cases studied, enabling individualized learning pathways, increasing accessibility for students with limited practicum opportunities, and facilitating objective evaluation of diagnostic skills.

**Conclusions:**

Integration of a digital simulator into the training process for speech therapists can significantly enhance the quality of education, strengthen diagnostic competencies, support personalized learning, and improve readiness for professional practice. The implementation of such tools contributes to addressing an important social objective — providing high-quality speech therapy services for children with speech disorders, and, consequently, preserving and promoting the mental health of these children.

**Disclosure of Interest:**

None Declared

